# Prion Remains Infectious after Passage through Digestive System of American Crows (*Corvus brachyrhynchos*)

**DOI:** 10.1371/journal.pone.0045774

**Published:** 2012-10-17

**Authors:** Kurt C. VerCauteren, John L. Pilon, Paul B. Nash, Gregory E. Phillips, Justin W. Fischer

**Affiliations:** United States Department of Agriculture, Animal and Plant Health Inspection Service, Wildlife Services, National Wildlife Research Center, Fort Collins, Colorado, United States of America; Ohio State University, United States of America

## Abstract

Avian scavengers, such as American crows (*Corvus brachyrhynchos*), have potential to translocate infectious agents (prions) of transmissible spongiform encephalopathy (TSE) diseases including chronic wasting disease, scrapie, and bovine spongiform encephalopathy. We inoculated mice with fecal extracts obtained from 20 American crows that were force-fed material infected with RML-strain scrapie prions. These mice all evinced severe neurological dysfunction 196–231 d postinoculation (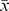 = 198; 95% CI: 210–216) and tested positive for prion disease. Our results suggest a large proportion of crows that consume prion-positive tissue are capable of passing infectious prions in their feces (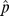 = 1.0; 95% CI: 0.8–1.0). Therefore, this common, migratory North American scavenger could play a role in the geographic spread of TSE diseases.

## Introduction

Transmissible spongiform encephalopathies (TSE) are most likely caused by pathogenic isoforms (PrP^Res^) of prion proteins [1] that naturally occur across many classes of animals, including mammals and birds [2]. A number of livestock and wildlife species in North America are susceptible to TSE diseases. Mule deer (*Odocoileus hemionus*), white-tailed deer (*O. virginianus*), elk (*Cervus elaphus*), and moose (*Alces alces*) are susceptible to chronic wasting disease (CWD); domestic sheep and goats are susceptible to scrapie; and domestic cattle are susceptible to bovine spongiform encephalopathy (BSE) (although this disease is rare in North America [3]). These TSE diseases are always fatal to infected animals, and upon death, carcasses allowed to remain in the environment can be scavenged by an array of avian and mammalian scavengers [4].

Mechanisms for the spread of TSE in wild and domestic ungulates are incompletely understood. We hypothesized that avian scavengers have potential to translocate PrP^Res^ in their feces. American crows (*Corvus brachyrhynchos*) are significant avian scavengers of deer carcasses [4], they are migratory, and their overall range [5] includes most areas where TSE diseases occur in North America [6]. Crows forage in groups, traveling up to 80 km/d from communal roosts [5]. Thus, crows have opportunity to encounter PrP^Res^-infected carcasses, consume infected tissue, and move long distances before depositing feces. Once in the soil, PrP^Res^ may persist >2 years [7], [8], potentially enabling increased site contamination over time. For example, residual contamination of soil with PrP^Res^ caused recurrence of CWD in confined mule deer in Colorado [7] and lateral transmission via environmental contamination is likely an important route of infection [9].

Insects [10], [11], poultry [12], and scavengers, including crows [4], have been suggested as passive carriers or dispersers of infectious prions. We found no studies that evaluated passage of PrP^Res^ through avian digestive systems, though several studies have evaluated resistance of PrP^Res^ to mammalian digestive fluids. Ruminant digestive fluids used during in-vitro trials have shown substantial [13], [14] to no reduction [15] in Western blot signal after incubation periods of approximately 13–24 h. Shorter incubation times (15–210 min) resulted in intermediate levels of Western blot signal loss [16]. Studies that investigated effects on PrP^Res^ from full passage through rodent digestive systems found scrapie and BSE PrP^Res^ present in mouse feces [17] and scrapie PrP^Res^ in hamster feces (ca. 5% of original dose excreted 24 h postinoculation) [18]. Thus, it appears that mammalian digestive fluids and processes can reduce PrP^Res^ concentration but are unlikely to eliminate it.

Proteolysis occurs in the avian digestive system due to the presence of hydrochloric acid (HCl) and the proteolytic enzymes pepsin, trypsin, chymotrypsin and various peptidases [19], [20]. Although experimentally induced hypoacidity was associated with reduced scrapie infection rates in mice [21], it is unlikely that gastric HCl would fully degrade PrP^Res^ in the crow digestive system given extreme temperature and concentration required [22] and mild conditions present in the avian gut [19], [23]. Although early investigations suggested that trypsin reduced scrapie titer under certain circumstances [1], [24], subsequent studies found pepsin and trypsin were not effective for reducing infectivity of scrapie and BSE PrP^Res^ [25] or variant Creutzfeldt-Jakob disease PrP^Res^ [26]. Thus, there is little evidence to suggest that the crow digestive system would eliminate PrP^Res^ infectivity prior to excretion of feces. Similar arguments can be made for nonruminant mammals because of similarities in endogenous enzymes in vertebrate digestive systems [27], yet PrP^Res^ was substantially reduced by passage through hamster digestive systems [18].

Little is known about effects of avian digestive systems on infectivity of PrP^Res^. As a first step in understanding the potential role of avian scavengers in TSE transmission, we tested the hypothesis that readily available mouse-adapted scrapie PrP^Res^ can remain infectious after passage through the digestive tract of crows. Results of our study support this hypothesis.

## Materials and Methods

We evaluated infectivity of the RML Chandler strain (RML) of mouse-adapted scrapie [28] (obtained from Rocky Mountain Laboratories, Hamilton, MT) after passage through digestive systems of crows. Crows were captured during winter in central Oklahoma, USA. We used mouse-brain source material from uninfected (normal) and terminally ill RML-infected C57BL/6 mice (Hilltop Lab Animals, Scottsdale, PA; this strain used throughout study). We separately pooled and homogenized infected and normal mouse brains and diluted portions of each homogenate 1∶10 w/v in sterile phosphate-buffered saline (SPBS). We estimated passage time through the alimentary canal by gavaging 1 crow (not part of the experimental group) with 5 ml of whole egg mixed with blue dye; by 4 h postgavage all stained feces had been excreted. We withdrew feed (but not water) from study crows approximately 17 h pregavage. We randomly allocated 25 crows to treatment groups and gavaged each crow with 5 ml of either PrP^Res^-infected (n = 20) or normal (n = 5) mouse-brain homogenate diluted 1∶10 w/v in SPBS (Table 1). We then transferred each crow to an individual single-use cage. At 4 h postgavage, we collected and pooled all feces within each cage. We homogenized crow-specific pooled feces and gamma irradiated them at 24,000 Gy to destroy viruses and microbes. For each crow, we then diluted a 500 µl sample of fecal homogenate in SPBS to a total volume of 10 ml, centrifuged it for 15 min at 13,730 m/s^2^, and extracted the supernatant for use as inoculum for mice. We removed solids to minimize risk of toxicity to mice from uric acid contained in bird feces. Crows were not held or examined after collection of fecal samples.

**Table 1:** Experimental design used to estimate proportion of crows able to pass infectious RML scrapie prion (PrP^Res^) in feces (numbers of animals).

Treatment group^A^	Crows	Mice^B^
CF+	20	100
CF−	5	25
MB+	0	10
MB−	0	5

A Mice intraperitoneally inoculated with gamma-irradiated crow fecal (CF) extract from crows gavaged with PrP^Res^ (+) or control (−) mouse brain homogenate; additional control mice were inoculated with mouse-brain homogenate with (MB+) or without (MB−) PrP^Res^.

B Five mice were randomly allocated to each crow and housed together in 1 cage postinoculation. Additional control mice were allocated randomly to MB treatment groups and 5 mice/treatment group were housed together in 1 cage postinoculation.

We randomly allocated 5 mice/crow to treatment groups (Table 1). Mice received crow-specific fecal supernatant from PrP^Res^ or control crows (CF+ and CF− groups, respectively), or PrP^Res^-infected or normal mouse brain homogenate diluted to 1∶100 w/v in SPBS (MB+ and MB− groups, respectively). We intraperitoneally inoculated each mouse with 1 ml of either crow fecal supernatant or diluted mouse brain homogenate.

All 5 mice/crow, or 5 mice/MB treatment group, were caged together under biosafety level 2 conditions. We monitored mice daily until all those in PrP^Res^ treatment groups expressed clinical symptoms of mouse scrapie and were thereafter euthanized. Remaining mice were monitored every 2 d until study termination at 365 d postinoculation (dpi). We scored mice for each of 6 clinical symptoms of mouse scrapie (kyphosis, ataxia, stiff tail, lack of grooming, emaciation, and lethargy), where 0 = none visible, 1 = moderate, and 2 = severe. We euthanized mice when total daily scores reached ≥8 for 1 d, ≥6 continuously for 3 d, or at 365 dpi. Brains were immediately harvested and stored at −70°C for analysis. Samples from harvested brains (1∶10 w/v homogenate) were tested at Colorado State University's Veterinary Diagnostic Laboratory for PrP^Res^ using the ELISA-based Bio-Rad TeSeE BSE rapid assay (Bio-Rad Laboratories, Hercules, CA, USA) to confirm scrapie diagnosis.

We used exact methods [29] to estimate a 95% confidence interval (CI) on the proportion of crows able to excrete infectious prions in feces (SAS PROC FREQ [30]). We used Fisher's exact test, due to low count (i.e., 2) in 1 cell of the 2×2 contingency table, to evaluate whether early death (≤3 dpi) was associated with source of CF inoculum (PrP^Res^ or control). We estimated means and 95% CI for incubation time or time-to-death (contingent on surviving >3 dpi) for CF+ and MB+ mice using general linear mixed modeling [31], where cage was a random effect to account for clustering of mice within cages (SAS PROC GLIMMIX [30]). Traditional time-to-event (or survival) analyses were not required for CF+ and MB+ mice because none were censored >3 dpi. As most CF− mice were censored at study termination, we tested for equality of survival functions between CF+ and CF− using the log-rank test (SAS PROC LIFETEST [30]).

### Ethics Statement

The Institutional Animal Care and Use Committee of the United States Department of Agriculture, Animal and Plant Health Inspection Service, Wildlife Services, National Wildlife Research Center approved all procedures used in this study (QA-1406).

## Results

**Figure 1:**
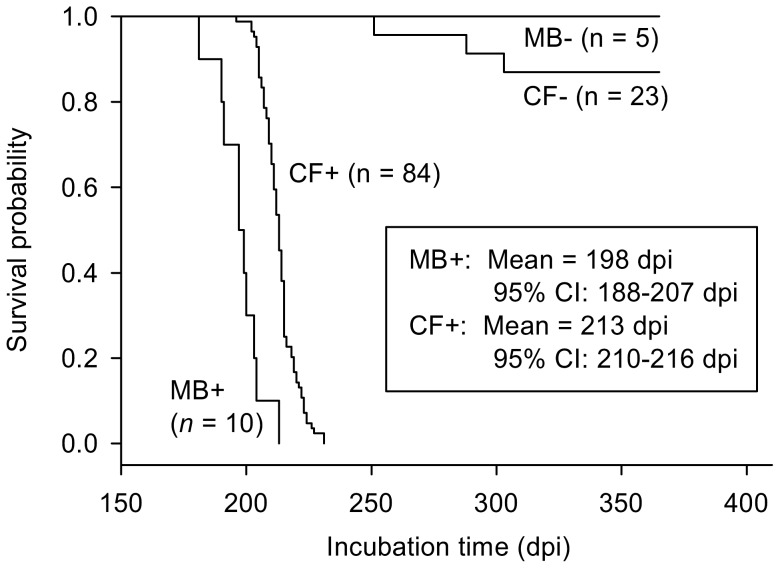
Survival functions for treatment groups of mice. Twenty-five crows were fed infected (PrP^Res^) or normal (control) mouse brain homogenate. Five mice/crow were subsequently inoculated with crow fecal extract from PrP^Res^ (CF+) or control (CF−) crows. Additional control mice were inoculated with mouse-brain homogenate with or without PrP^Res^ (MB+ and MB−, respectively). Sample sizes reflect early deaths of 16 mice ≤3 d postinoculation (dpi). Mean and interval estimates of survival time for MB+ and CF+ groups showed these groups were significantly different, indicating different dose levels of PrP^Res^ in crow fecal extracts compared to mouse brain homogenate. Time to death was significantly longer for CF− than for CF+ mice (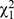 = 71.0, *p*<0.0001). Because all mice exposed to CF+ extracts died of transmissible spongiform encephalopathy (given survival >3 dpi), all 20 crows gavaged with PrP^Res^-infected mouse brain homogenate passed infectious doses of PrP^Res^ to mice via fecal extracts.

All 20 crows gavaged with scrapie-infected mouse brain transmitted PrP^Res^ to mice via fecal inoculum (estimated proportion: 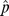 = 1.00, CI: 0.83–1.00). Sixteen mice from CF+ and 2 from CF− groups died ≤3 d postinoculation (likely from residual uric acid toxicity; Table 2). No early deaths occurred in MB groups and estimated probabilities of early death were not statistically different between CF+ (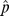 = 0.16) and CF− (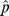 = 0.08) mice (Fisher's exact *P* = 0.524). After these early deaths, 2 crows were represented by only 1 mouse/crow and all other crows were represented by 3–5 mice/crow. Surviving mice appeared healthy until onset of clinical symptoms of mouse scrapie. Based on scoring for multiple clinical symptoms, we euthanized mice in MB+ and CF+ groups 181–231 dpi (Fig. 1). These mice subsequently tested positive for PrP^Res^ (Table 2). On average, MB+ mice had shorter incubation times (by 15 d) than CF+ mice (Fig. 1). We observed no clinical symptoms in MB− or CF− control mice. All MB− mice lived to study termination at 365 dpi, though 3 CF− mice died at 251–303 dpi. Time to death was significantly longer for CF− than for CF+ mice (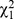 = 71.0, *p*<0.0001). One of these CF− mice (251 dpi) tested positive for PrP^Res^. This unexpectedly positive mouse was inoculated directly after 5 MB+ mice and may have been inadvertently exposed to PrP^Res^-positive material.

**Table 2:** Numbers of mice by treatment group that suffered early inoculation-related death, exhibited clinical symptoms of prion disease, and tested positive for scrapie prion (PrP^Res^) by ELISA^A^.

Treatment group^B^	Early death^C^	Clinical disease^D^	PrP^Res^ detected
CF+	16 (100)	84 (84)	84 (84)
CF−	2 (25)	0 (23)	1 (23)
MB+	0 (10)	10 (10)	9 (9)
MB−	0 (5)	0 (5)	0 (4)

A Numbers in parentheses indicate sample size.

B Mice intraperitoneally inoculated with gamma-irradiated crow fecal (CF) extract from crows gavaged with PrP^Res^ (+) or control (−) mouse brain homogenate; additional control mice were inoculated with mouse-brain homogenate with (MB+) or without (MB−) PrP^Res^.

C Mice that died ≤3 d postinoculation, presumably from fecal uric acid toxicity. These mice were removed from the data set.

D Mice that achieved a minimum threshold score, based on multiple symptoms such as kyphosis, ataxia, stiff tail, lack of grooming, emaciation, and lethargy, demonstrating strong clinical evidence of prion disease.

## Discussion

We tested the hypothesis that PrP^Res^ would remain infectious after passage through the digestive tract of crows. After inoculation with fecal supernatant from crows gavaged with PrP^Res^-infected material, we observed clinical disease and obtained positive results from ELISA in all 84 CF+ mice that survived >3 dpi. Thus, we confirmed passage of infectious PrP^Res^ through all 20 crows gavaged with infected material. We conclude that 83–100% of crows from the population we sampled can excrete infectious RML PrP^Res^ in feces under conditions similar to those in our study.

The MB+ mice developed clinical scrapie 15 d earlier than CF+ mice indicating inoculated dose of PrP^Res^ infectivity was likely lower for CF+ mice. We inoculated MB+ and MB− mice to demonstrate that brain source materials were infectious or not infectious, respectively, not to serve as standards for titer assessment. However, comparison with unpublished titration results from intraperitoneal inoculation of RML mouse scrapie into C57BL10 mice (Ann Ward and Sue Priola, Rocky Mountain Laboratories, personal communication) suggest MB+ mice received approximately 10-times more infectivity than CF+ mice. Dilutions of brain and fecal material with SPBS (see Methods) indicate that the amount of infectivity inoculated into MB+ mice would have been about double that of CF+ mice, assuming no influence on concentration of infectivity due to passage or centrifuge processing. It is reasonable to expect some loss of infectivity after removing solids from diluted crow feces by centrifugation. It is also possible that some degradation or absorption of infectivity occurred during passage through crow alimentary tracts.

Our study clearly shows that RML PrP^Res^ can persist after passage through the crow alimentary tract. As there is variability in resistance of different strains of PrP^Res^ to degradation [32]–[36], we cannot definitively state that passage of strains of concern would occur. However, RML PrP^Res^ has been shown more sensitive to degradation than TSE field isolates after 4 h exposure to enzymatic digestion [36]. Therefore, results of our study likely understate potential for prion passage through the alimentary canal of crows. Further experimental trials involving TSE prions obtained from ovine, bovine, and cervine carcasses would be required to definitively evaluate passage of natural TSEs through digestive systems of scavengers and predators. Other additional research topics could include in-vitro evaluation of PrP^Res^ degradation in crow digestive fluids; effects of solid, semisolid, and liquid delivery of infective materials on passage rate and residual infectivity in feces; postexcretion continued enzymatic and bacterial degradation of infectivity in feces; infectivity of feces excreted >4 h postgavage; susceptibility of crows to TSE disease and potential for postinfection shedding of PrP^Res^ in feces.
